# Effects of rhBMP-2-loaded hydroxyapatite granules/beta-tricalcium phosphate hydrogel (HA/β-TCP/hydrogel) composite on a rat model of caudal intervertebral fusion

**DOI:** 10.1038/s41598-022-12082-y

**Published:** 2022-05-12

**Authors:** Shinichi Nakagawa, Rintaro Okada, Junichi Kushioka, Joe Kodama, Hiroyuki Tsukazaki, Zeynep Bal, Daisuke Tateiwa, Yuichiro Ukon, Hiromasa Hirai, Takahiro Makino, Shota Takenaka, Seiji Okada, Takashi Kaito

**Affiliations:** 1 Department of Orthopaedic Surgery, Osaka University Graduate School of Medicine, 2-2 Yamadaoka, Suita, Osaka 565-0871 Japan; 2 Department of Orthopaedic Surgery, Osaka General Medical Center, Osaka, Osaka Japan; 3 Department of Orthopaedic Surgery, Kansai Rosai Hospital, Amagasaki, Hyogo Japan

**Keywords:** Medical research, Materials science

## Abstract

The effects and inflammation-related side effects of bone morphogenetic protein (BMP)-2 on posterior lumbar interbody fusion are controversial. One of the potential causes for the inconsistent results is the uncontrolled release of BMP-2 from the collagen carrier. Therefore, BMP delivery systems that support effective bone regeneration while attenuating the side effects are strongly sought for. We developed NOVOSIS putty (NP), a novel composite material of hydroxyapatite (HA), beta-tricalcium phosphate (β-TCP)/hydrogel, and BMP-2, which can sustainably release BMP-2 over 2 weeks. This study was aimed at comparing the effects and side effects of NP and collagen sponge (CS) containing BMP-2 using a rat caudal intervertebral fusion model. The fusion rates of NP with low and high doses of BMP-2 were significantly higher than those of an iliac bone (IB) graft, but those of CS with low and high doses of BMP-2 were not different from those of the IB graft. Furthermore, the incidences of ectopic bone formation and soft tissue swelling were significantly lower in the NP group than in the CS group. The HA/β-TCP/hydrogel carrier enabled superior bone induction with low-dose BMP-2 and decreased the incidence of side effects caused by high-dose BMP-2 vis-à-vis the collagen carrier.

## Introduction

After the approval of recombinant human bone morphogenetic protein-2 (rhBMP-2) as a bone graft substitute for anterior lumbar intervertebral fusion by the Food and Drug Administration in 2002^1,2^, its use, including the off-label use, has been reported for approximately 30% of lumbar spine fusion surgeries in the United States^2^. However, adverse events related to the use of the supraphysiological doses (in the order of milligrams) of rhBMP-2, such as soft tissue swelling, local inflammation, osteolysis, ectopic bone formation, retrograde ejaculation, and radiculitis, prevent its widespread adoption^3–5^. Thus, there is a need for an efficient drug delivery system for rhBMP-2 to mitigate these adverse events by enabling efficient bone formation with low-dose rhBMP-2.

Several carriers and drug delivery systems have been developed for rhBMP-2 to date, including collagen, hydroxyapatite (HA), beta-tricalcium phosphate (β-TCP), synthetic polymers, and hydrogels^6–11^. HA can provide high mechanical strength, but its biodegradability is limited^12^. β-TCP is biodegradable, but its mechanical strength is low^13^. Synthetic polymers and hydrogels enable sustained release of rhBMP-2^14–16^; however, a single application of synthetic polymers causes difficulties with molding and handling during surgery. Therefore, we combined multiple carriers (HA/β-TCP microsphere/poloxamer 407 hydrogel) and created a novel composite biomaterial for rhBMP-2 (Novosis putty® [NP], CGBio Co., Seoul, Korea) to compensate for their shortcomings and take advantage of their strengths. In the NP, HA and β-TCP are expected to provide scaffolds for new bone formation for long and short periods, respectively, and the hydrogel would enable sustained release of rhBMP-2 over 3 weeks^17–19^.

The purpose of this study was to elucidate the effects of NP as a carrier for rhBMP-2 in bone formation and to compare the adverse events of NP with those of a collagen sponge (CS) carrier using a rat caudal intervertebral fusion model.

## Results

### Fusion rates

Rats were divided into the following five groups based on the grafting materials (allogenic iliac bone only [IB group], CS soaked with rhBMP-2 [CS group], and HA/β-TCP/hydrogel containing rhBMP-2 [NP group]) and the dosage of rhBMP-2 (3 µg of rhBMP-2 [low BMP] or 10 µg of rhBMP-2 [high BMP]) (n = 8 for each group).

**Figure 1:**
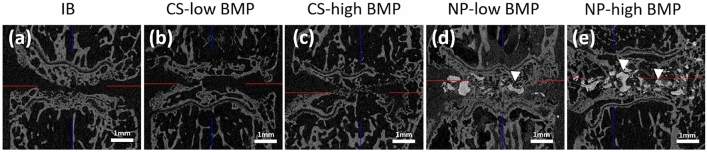
Ex vivo micro-CT images of treated segments. Representative coronal micro-CT images of treated intervertebral discs in each group. In the IB group, the grafted bone almost disappeared, and no bridging new bone formation was observed (**a**). In the CS-low and -high BMP groups, new bone formation that partly bridged the intervertebral disc space was observed, but the bone volume and density were low (**b**,**c**). In the NP-low and NP-high groups, the intervertebral disc space was occupied by dense new bone formation with a small amount of HA particles (**d**,**e**).

Spinal segments harvested at 6 weeks postoperation were scanned using high-resolution ex vivo micro-computed tomography (micro-CT). Intervertebral fusion was defined as bridging of bone formation in both the coronal and sagittal images of the intervertebral disc space. The fusion rates at the intervertebral disc space were 25% in the IB group, 50% in the CS-low BMP group, 62.5% in the CS-high BMP group, and 87.5% in both NP-low and -high BMP groups, as determined using micro-CT. The fusion rate in both the NP groups was significantly higher than that in the IB group (*p* = 0.04). In contrast, the fusion rates in the CS group did not differ from those in the IB group (*p* = 0.21) **(**Fig. 1**)**.

### Time-dependent changes in the bone during the premortal period determined using micro-CT

**Figure 2:**
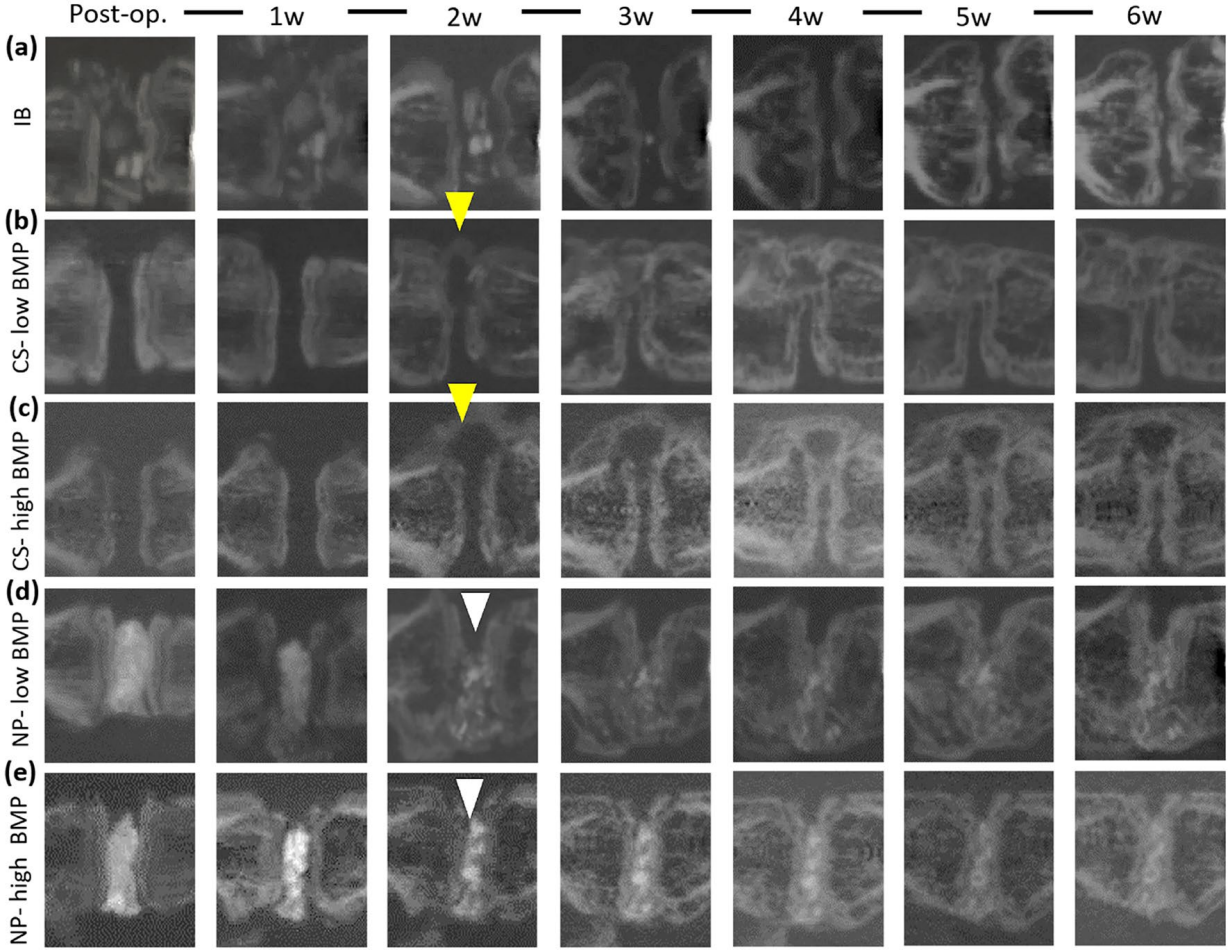
Temporal in vivo micro-CT images of the treated segments. In the IB group, the grafted bone was absorbed over time and almost disappeared at 6 weeks postoperation (**a**). In the CS group, new bone formation outside the disc space was observed at 2 weeks postoperation. The amount of new bone formation at the intervertebral disc space was limited until 6 weeks postoperation (**b**,**c**). In the NP groups, the density of HA and β-TCP decreased over time. New bone formation, limited to the disc space, was observed at 2 weeks postoperation, and the density of the new bone increased over time until 6 weeks postoperation (**d**,**e**).

In vivo micro-CT was performed immediately after surgery, 2 days postoperation, and every week after surgery until euthanasia (6 weeks). In the IB group, the density of the implanted IB decreased with time and the bone almost disappeared at 6 weeks postoperation. In both the CS groups, newly formed bone appeared outside the intervertebral disc space at 2 weeks postoperation, and new bone formation toward the intervertebral disc space was observed; however, the amount of bone formation at the disc space was small even at 6 weeks postoperation. In contrast, in both the NP groups, new bone formation was observed only in the intervertebral space, and the density of the new bone was increased (Fig. 2).

### Evaluation of adverse events at the surgical sites

**Figure 3:**
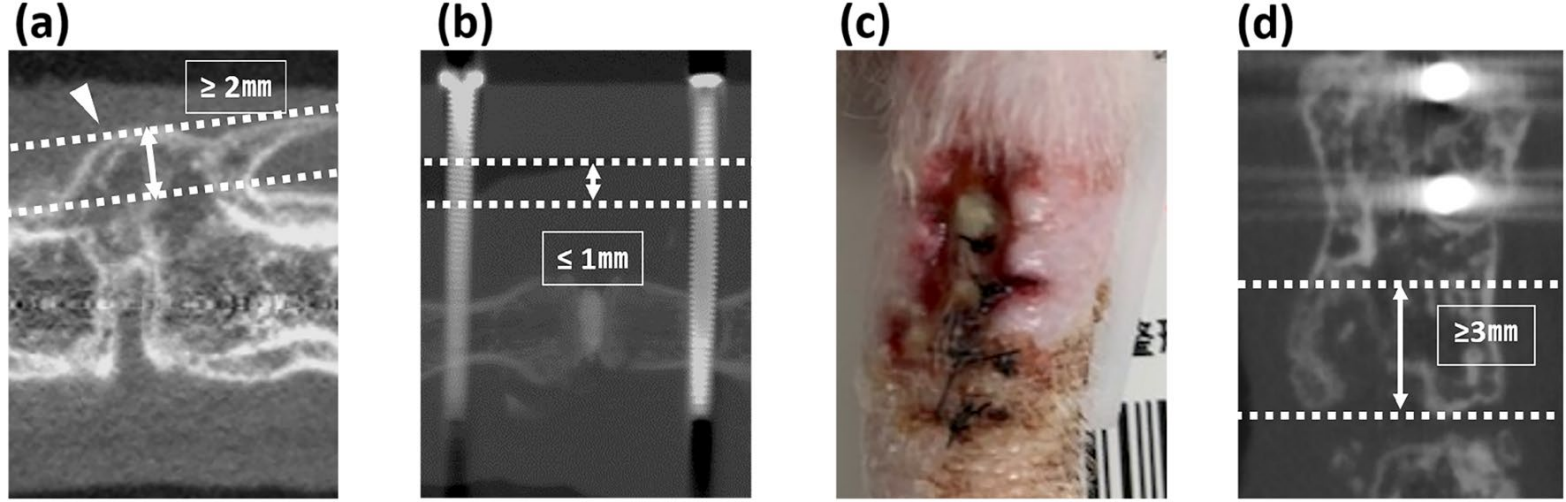
Representative images of each event. Operative site (**c**), and in vivo micro-CT images (**a**,**b**,**d**) as representative images of adverse events. (**a**) Ectopic bone formation outside the intervertebral disc space (white arrowhead). (**b**) Soft tissue swelling. (**c**) Delayed wound healing. (**d**) Osteolysis. Double headed arrows represent the measured distances for each event.

The incidence of adverse events, including delayed wound healing, soft tissue swelling, ectopic bone formation, and osteolysis, was observed as follows. Ectopic bone formation was diagnosed when bone formation outside the intervertebral disc space greater than 2 mm was observed using in vivo micro-CT (Fig. 3a). In soft tissue swelling, the distance between the skin surface and plate, measured using in vivo micro-CT, decreased to less than 1 mm on day 2 postoperation (Fig. 3b). Delayed wound healing was diagnosed when the operative wound did not heal within 2 weeks (Fig. 3c). Osteolysis of endplates was diagnosed when erosion of the bony endplate > 3 mm was observed using in vivo micro-CT (Fig. 3d). The total adverse event score (AES) was calculated by assigning one point for each adverse event.

The adverse events at the surgical site are summarized in **Table ****1**. The incidence of ectopic bone formation in the CS-high BMP group (87.5%) was significantly greater than that in the IB (0%; *p* = 0.01) and NP-high BMP (25%; *p* = 0.04) groups.

**Table 1:** Summary of the adverse events.

Group	Breakdown of adverse events			AES
	Incidence (%)			
	Ectopic bone	Soft-tissue swelling	Delayed wound healing	
IB	0^#^	12.5	12.5	0.25 ± 0.164^#^
CS-low BMP	50*	25	25	1.0 ± 0.378
CS-high BMP	87.5**	62.5*	37.5	1.88 ± 0.295*
NP-low BMP	12.5	25	12.5	0.5 ± 0.327^#^
NP-high BMP	25	37.5	12.5	0.75 ± 0.250^#^

*IB* iliac bone, *BMP* rhBMP-2, *CS* collagen sponge, *NP* Novosis putty, *AES* adverse event score.

*p < 0.05 (vs. IB group), **p < 0.001 (vs. IB group), #*p* < .05, (vs. CS-high BMP group).

The incidence of soft tissue swelling was 62.5% in the CS-high BMP group, 37.5% in the NP-high BMP group, 25% in both the CS-low BMP and NP-low BMP groups, and 12.5% in the IB group. The incidence of soft tissue swelling in the CS-high BMP group was significantly higher than that in the IB group (*p* = 0.03). The incidence of delayed wound healing was not different among the five groups. Osteolysis of the endplates was not observed in this study. The AES in the CS-high BMP group (1.88 ± 0.30) was higher than that in the NP-high BMP group (0.75 ± 0.25; *p* = 0.04).

### Swelling ratio at the surgical sites

**Figure 4:**
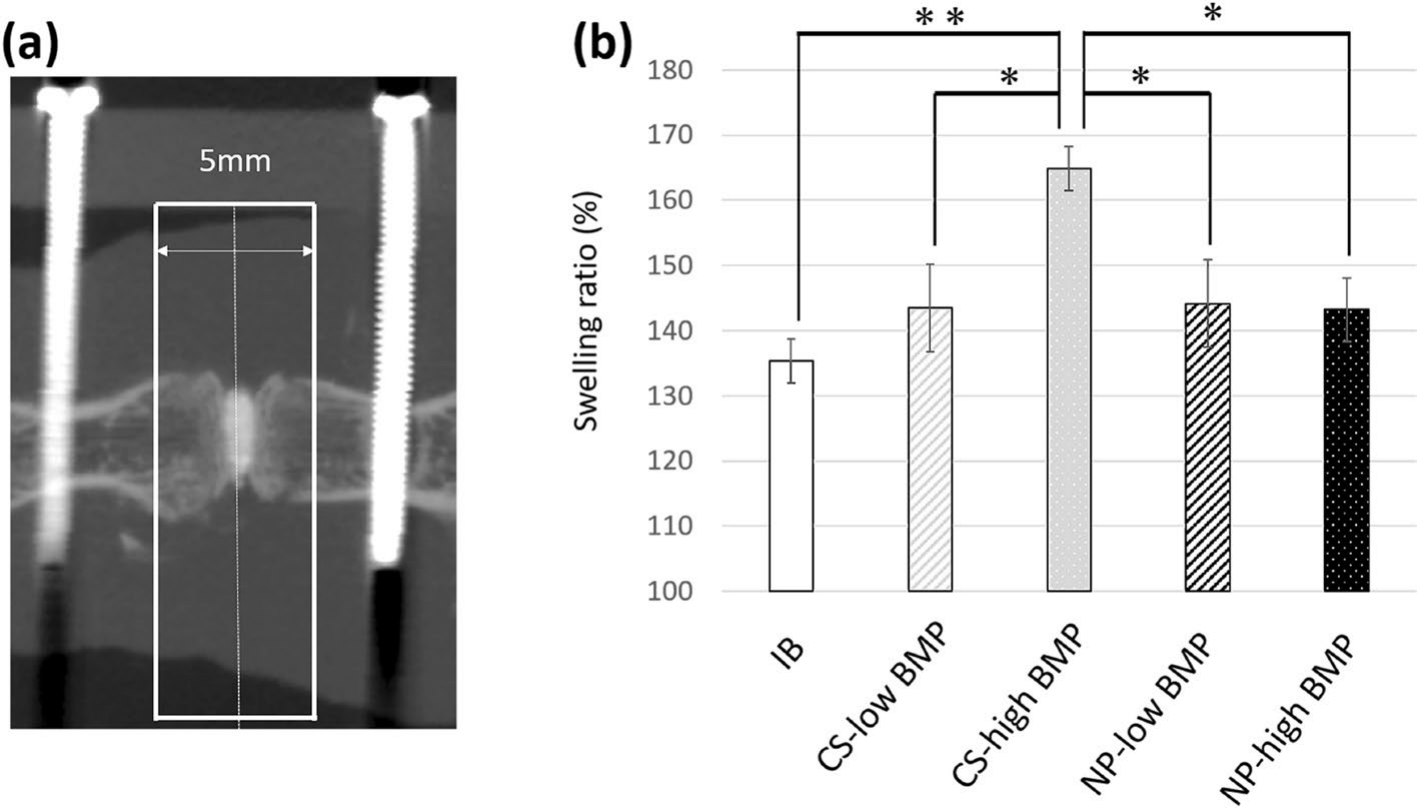
Quantification of surgical site swelling. (**a**) Region of interest for measurement of tissue volume. The dotted line denotes the middle of the intervertebral disc space. (**b**) Comparison of the swelling ratio among groups. The swelling ratio in the CS-high group was significantly greater than that in the other groups. *, *p* < 0.05; **, *p* < 0.001.

The ratio of soft tissue swelling was calculated by dividing the soft tissue volume (TV) of the surgical site on day 2 postoperation by the TV, 1 day before surgery (Fig. 4a). The swelling ratio in the CS-high BMP group (164.9 ± 3.4%) was significantly higher than that in all the other groups. The swelling ratio of the NP groups was not affected by the BMP-2 dose, in contrast to the CS groups, in which the swelling ratio was increased when combined with high-dose BMP-2 (Fig. 4b).

### Histological analysis

**Figure 5:**
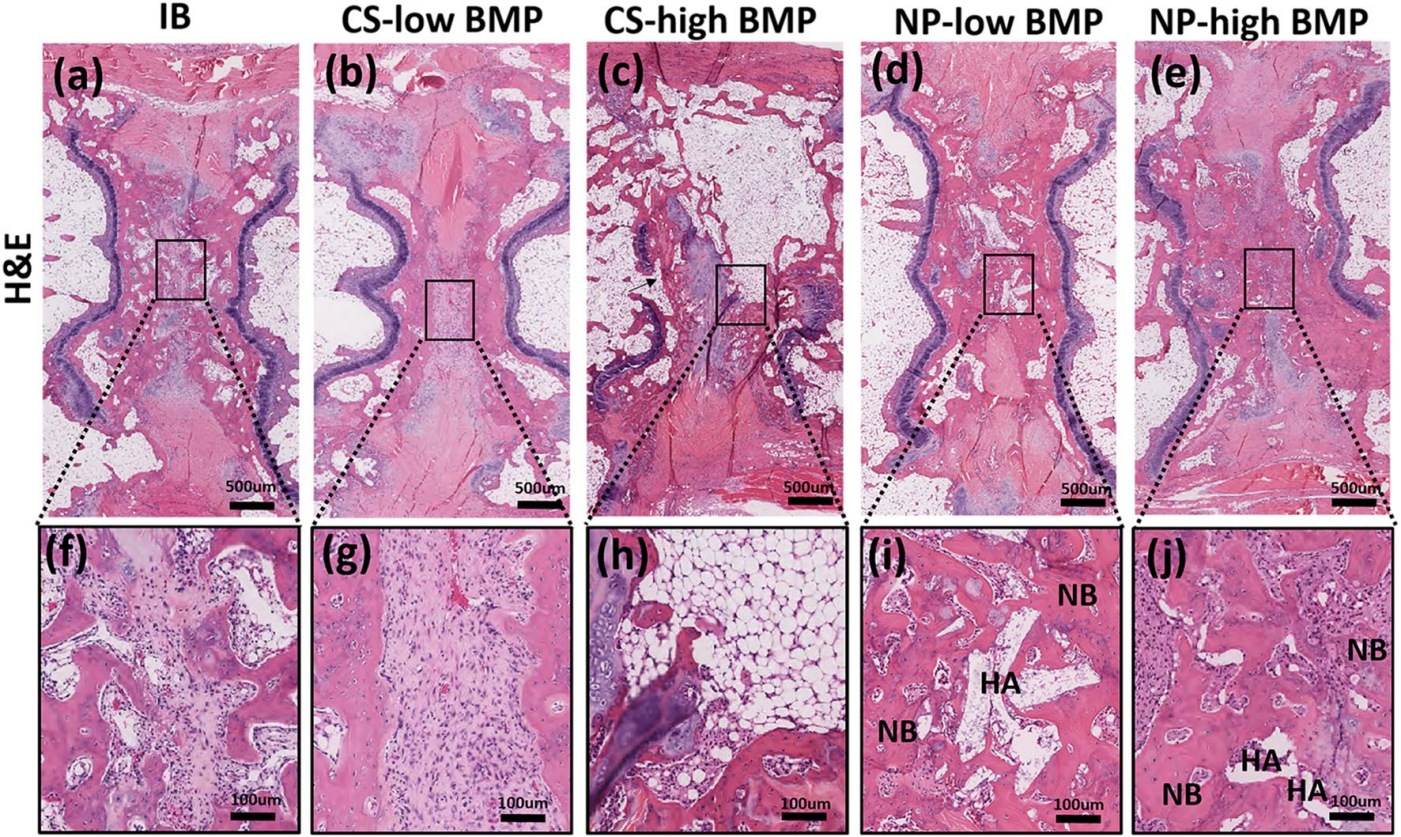
Histological images of treated segments at 6 weeks postoperation. Hematoxylin & eosin (H&E) staining of treated segments. Whole intervertebral disc space (**a**–**e**), Magnified central parts of the disc space (**f**–**j**). Fibrous tissue was present between adjacent endplates in the IB group (**a**,**f**) and in the CS-low BMP group (**b**,**g**). In the CS-high group, new bone formation that bridged the endplates was observed, but the new bone was predominantly composed of adipose tissue (**c**,**h**). Thick trabecular bone formation between the endplates was observed in both the NP groups (**d**,**e**). A small amount of HA granules remained inside the newly formed bone (**i**,**j**). NB, new bone; HA, hydroxyapatite.

In the IB group, the intervertebral disc space was predominantly composed of fibrocartilage tissue. In the CS-low BMP group, only a small amount of new bone formation was observed in the intervertebral disc space. In the CS-high BMP group, bridging of new bone formation between the endplates was observed, but the new bone tissue was predominantly composed of adipose tissue. In contrast, in both the NP groups, new bone formation bridging between the endplates was composed of thick trabecular bone. In the NP-low and NP-high groups, a small amount of HA granules remained in the intervertebral disc space, and some of the β-TCP microspheres were resorbed and replaced by new bone (Fig. 5).

### Quantification of the newly formed bone area in the interbody space

**Figure 6:**
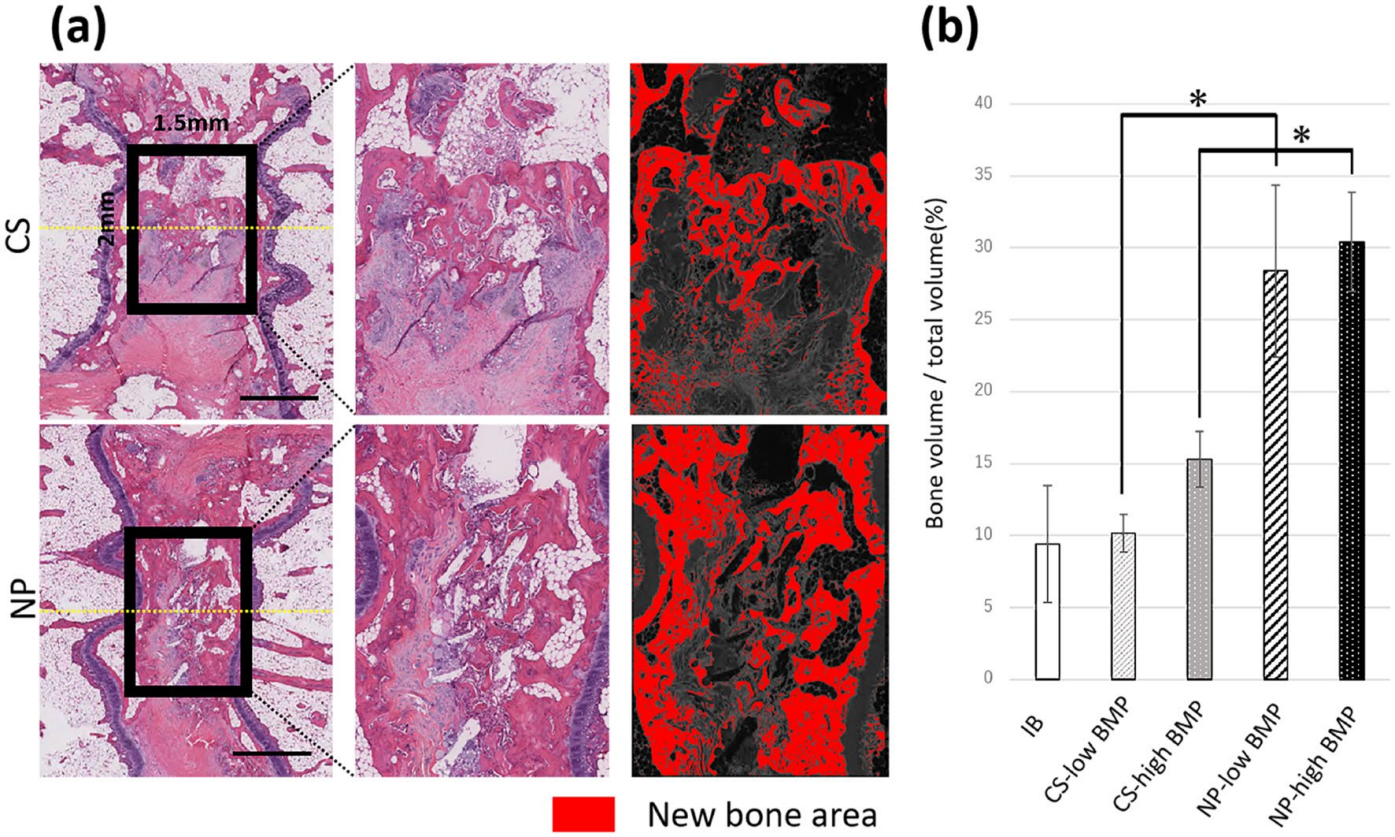
Quantification of the new bone area in the interbody space. (**a**) Comparison of new bone area inside the spinal fusion mass in histological sections. A 1.5 × 2 mm^2^ ROI (interbody space) was extracted from the newly formed fusion mass. The new bone area (red) was color-coded using the ImageJ software (version 1.52q, U. S. National Institutes of Health; https://imagej.nih.gov/ij/). (**b**) The percentage of the newly formed bone area in the ROI was significantly higher in the NP groups than in the CS groups (n = 8, *p < 0.03) (IB, 9.4%; CS-low BMP group, 10.1%; NP-low BMP group, 28.4%; CS-high group, 15.3%; NP-high group, 30.4%; data represent mean ± SD; *p < 0.03 by Tukey–Kramer test).

The percentage of new bone area in the region of interest (ROI) was significantly higher in the NP groups (NP-low BMP group, 28.4%; NP-high group, 30.4%) than in the CS group (CS-low BMP group, 10.1%; CS-high group, 15.3%; *p < 0.03; Fig. 6b).

## Discussion

In this study, the use of HA/β-TCP/hydrogel composite as a carrier for rhBMP-2 resulted in a superior bone fusion rate and a lower incidence of side effects compared with those observed following the use of CS in a rat model of caudal intervertebral fusion. In addition, new bone formation in the HA/β-TCP/hydrogel composite group was localized in the intervertebral disc space; in contrast, ectopic bone formation outside the intervertebral disc space was observed in the group treated with CS. Thus, the use of the HA/β-TCP/hydrogel composite enabled efficient and spatially controlled new bone formation by rhBMP-2.

The use of rhBMP-2 remains controversial because the efficacy of BMP-2 for high fusion rate and short operative time may be counteracted by potential side effects, such as ectopic bone formation, soft tissue swelling, local inflammation, osteolysis, and retrograde ejaculation^4,5^. In this study, using the HA/β-TCP/hydrogel as a carrier material for BMP-2, efficient and spatially controlled bone regeneration was achieved with fewer inflammation-related side effects. The characteristics of the HA/β-TCP/hydrogel composites mentioned below are expected to corroborate the results.

The first characteristic of the composite material is the sustained release of rhBMP-2. The BMP-2-related side effects have been reported to be dose dependent^20–22^. Therefore, the decrease in local rhBMP-2 concentration due to sustained release of rhBMP-2 can reduce the side effects. The β-TCP/hydrogel used in this study sustainably releases 20% of the total rhBMP-2 within 7 days, in contrast to the CS, which releases almost 100% of the total rhBMP-2 within 1 day^17^. In another study using the same HA/β-TCP/hydrogel composite, it was demonstrated that rhBMP-2 release within 7 days was 19.5% from the composite and 98.3% from the collagen carrier^19^. This sustained release of rhBMP-2 is supposed to contribute to the mitigation of BMP-2-related side effects and improvement in fusion rate by controlling the spatial spread of BMP-2 and inducing new bone formation for a long time. In fact, soft tissue swelling did not change between low dose (25%) and high dose (37.5%) of rhBMP-2 in the NP groups, in contrast to the CS groups, in which a significant increase in soft tissue swelling was observed in high-dose BMP group (62.5%) compared with that in low-dose BMP group (25%). Ectopic bone formation outside the intervertebral space was infrequent in the NP group (low BMP/high BMP, 12.5%/25%) compared to that in the CS group (low BMP/high BMP, 50%/87.5%). In addition, the quality of newly formed bone was superior in the NP group, in which abundant thick trabecular bone formation was observed, compared to that in the CS group, in which fatty bone marrow was predominant.

Another advantageous characteristic of the HA/β-TCP/hydrogel composite is the combination of two calcium phosphate scaffolds (HA and β-TCP) with different biodegradabilities. HA and β-TCP can provide biomechanical strength and moldability of the composite, and the low-biodegradable HA provides a long-term scaffold for bone formation^12^, and the highly biodegradable β-TCP microspheres can provide a space for new bone formation in addition to enhancing osteogenic cell differentiation^23–25^. Histological evaluation demonstrated that a small amount of HA granules remained in the intervertebral disc space at 6 weeks postoperation, but the majority of β-TCP was remodeled and regenerated into new bone (Fig. 5i,j).

Due to these advantages of NP by combining the traits of three materials (sustained release of rhBMP-2 by hydrogel, long-term scaffold for bone formation by HA and efficient replacement with new bone by biodegradable β-TCP), NP could compensate for the shortcomings of each material by utilizing the strengths of each and resulted in superior bone induction with low-dose BMP-2 and the decreased incidence of side effects caused by BMP-2.

This study has several limitations. First, the results for rodent models cannot be directly extrapolated to humans because of the different biomechanics of the spine between quadrupeds and bipeds. Second, no intervertebral fusion cages were applied to the intervertebral disc space in this study. The use of an intervertebral cage might increase the fusion rate in the IB group or rhBMP-2-loaded CS group. Finally, the release kinetics of rhBMP-2 (3 and 10 μg) from NP was not investigated. However, we have reported in vitro release kinetics of 4 μg—an amount not markedly different from that used in the present study—rhBMP-2 from NP^19^.

In conclusion, the HA/β-TCP/hydrogel composite enabled superior bone induction with a low dose of rhBMP-2 and reduced the incidence of side effects caused by high doses of rhBMP-2 compared with the collagen carrier in a rat model of caudal intervertebral fusion. The HA/β-TCP/hydrogel composite is a novel biomaterial for efficient bone regeneration using rhBMP-2.

## Methods

### Characterization of HA and β-TCP in HA/β-TCP/hydrogel composite

The HA/β-TCP/hydrogel biomaterial adopted in this study, which contained 40% HA and 60% β-TCP, was manufactured by CGBio Co., Ltd., Seoul, Republic of Korea. In addition to the assessment of their general appearance, the detailed microstructures of β-TCP/hydrogel and HA were determined using scanning electron microscopy (SEM; Hitachi S-4800, Japan) (Supplementary Fig. 1).

The HA granules, β-TCP microspheres, and hydrogel were manufactured by CGBio Co., Ltd., Seoul, Republic of Korea. The size of HA granules ranged from 3.0 to 6.0 mm, and they were characterized by approximately 70% porosity and 99% interconnectivity. The β-TCP microspheres produced using the spray-drying method were ~ 45–75 μm in size, with approximately 68% porosity. The hydrogel was composed of a polyethylene glycol (PEG)/polypropylene glycol (PPG)/PEG block copolymer and hydroxypropyl methylcellulose (HPMC) composite. The PEG/PPG/PEG block copolymer is thermosensitive and is liquid at low temperature and can be mixed homogeneously, while it is a gel at body temperature and can be shaped into a desired form. The HPMC composite is viscoelastic, which is helpful in setting a certain shape for the final injected form, with increased resistance against external stress.

### Animals and experimental groups

The Animal Experimental Committee of Osaka University Graduate School of Medicine approved all animal studies (approval number: 28-076-014), which were performed in accordance with the ARRIVE guidelines and the National Institutes of Health Guide for the Care and Use of Laboratory Animals^26^. Forty 8-week-old male Sprague–Dawley (SD) rats (Charles River Laboratories, Japan Inc., Kanagawa, Japan) were used in this study. The rats were divided into the following five groups based on the grafting materials: allogenic iliac bone only (IB group), CS soaked with rhBMP-2 (CS group), and HA/β-TCP hydrogel containing rhBMP-2 (NP group), as well as the dosage of rhBMP-2 (3 µg of rhBMP-2 [low BMP] or 10 µg of rhBMP-2 [high BMP]) (n = 8 for each group). The dosage of rhBMP-2 was decided based on previous reports in which the same caudal intervertebral fusion model was used^27^. Volumetric comparison of rat and human intervertebral disc space revealed that 5 μg of rhBMP-2 in rats corresponds to 1.5 mg of rhBMP-2 in humans.

### Preparation of grafting materials

#### Allogenic iliac cancellous bone

Allogenic iliac cancellous bone was harvested from a donor SD rat immediately before surgery. The volume of iliac bone grafting for each disc space was set to 60 mm^3^ (0.05 mg).

#### Collagen sponge containing rhBMP-2

CS (CollaCote; Zimmer Dental, FL, USA) was cut into 5 mm × 5 mm ×  3 mm (60 mm^3^) sections. Next, 3 μg or 10 μg of *Escherichia coli*-derived rhBMP-2 (Daewoong Pharmaceutical Co., Ltd., Seoul, Korea)^28^ was dissolved in phosphate-buffered saline and impregnated into the CS and then freeze-dried (Free Zone 2.5, Labconco Co., Ltd., Kansas city, MO, USA).

#### HA/β-TCP/hydrogel composite

**Figure 7:**
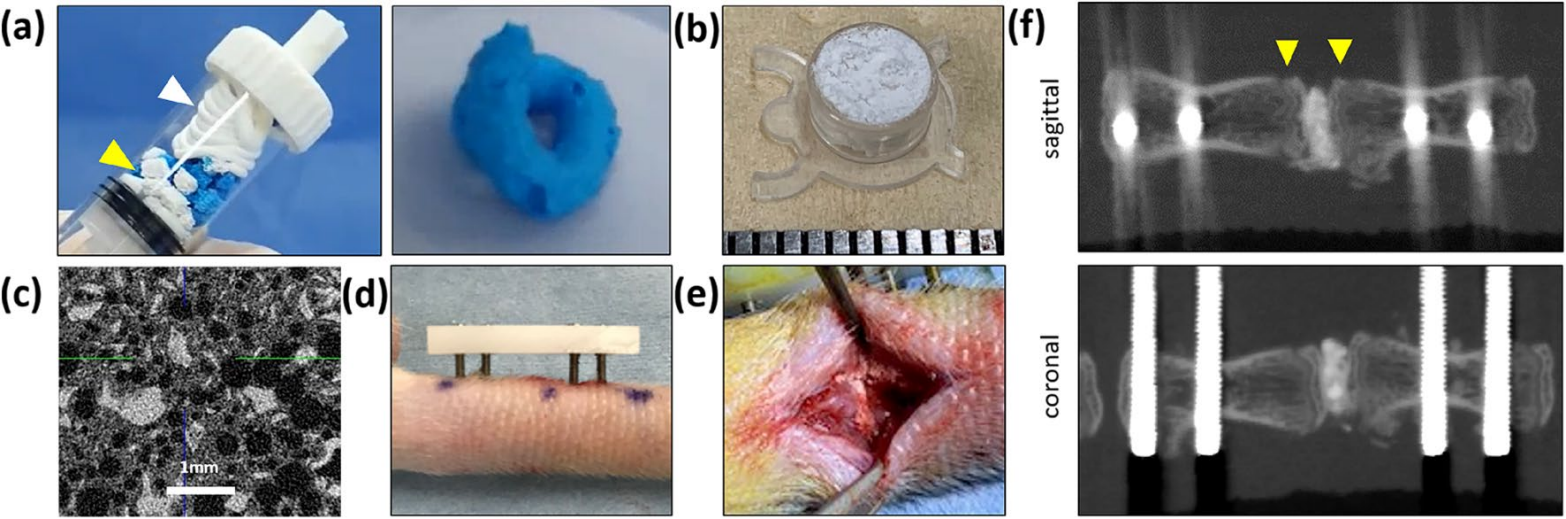
Process for the production of HA/β-TCP/hydrogel, operative photograph, and postoperative micro-computed tomography images. Preparation of HA/β-TCP/hydrogel loaded with rhBMP-2 (**a**), HA/β-TCP/hydrogel used for insertion (**b**), ex-vivo μ-CT image of HA/β-TCP/hydrogel (**c**), caudal intervertebral fusion (**d**), and disc space after implantation of HA/β-TCP/hydrogel (**e**). Postoperative sagittal (f, upper) and coronal (f, lower) views of micro-CT, with yellow arrowheads indicating the surface of the bony endplate in the sagittal view.

Materials for production of NP, including HA granules, β-TCP microsphere/poloxamer 407 hydrogel, and rhBMP-2, were kindly provided by CGBio Co., Ltd.^28^. The hydrogel contained poloxamer 407, which is a biodegradable and biocompatible polymer that has been applied in various fields as a biomaterial^29,30^. The hydrogel was mixed with β-TCP microspheres at a 1:1 weight ratio. Next, HA granules were soaked with rhBMP-2, crushed into small pieces, and mixed with the β-TCP/ hydrogel at a 3:2 weight ratio in a special mixing syringe to form homogeneous clay-like composites (Fig. 7a). The composites were molded in a container to a volume of 60 mm^3^ (0.05 mg) before implantation (Fig. 7b,c).

#### Rat caudal intervertebral fusion

All rats were anesthetized using a combination of 0.15 mg/kg medetomidine (Nippon Zenyaku Kogyo Co., Ltd., Fukushima, Japan), 2.0 mg/kg midazolam (Astellas Pharma, Inc., Tokyo, Japan), and 2.5 mg/kg butorphanol (Meiji Seika Pharma Co., Ltd., Tokyo, Japan). Polyoxymethylene plates (23 mm long × 6 mm wide × 3 mm high; Matec Co., Ltd., Osaka, Japan) were fixed to the sixth and seventh caudal vertebrae through the skin using four stainless steel screws (two screws for each vertebra, φ1.2 mm × 14 mm; Matsumoto Industry Co., Ltd., Chiba, Japan). The distance between the plates and the skin surface was maintained at 3 mm at the time of plate fixation (Fig. 7d).

Subsequently, a 10 mm dorsal midline incision was made to expose the intervertebral disc. The posterior one-third of the annulus fibrosus and total nucleus pulposus were removed, and then the cartilaginous endplates were detached and excised using a small raspatory and rongeur to avoid damage to the bony endplates^27^. After irrigation with saline, grafting materials (60 mm^3^ of IB, CS, or NP) were implanted into the intervertebral disc space (Fig. 7e). The screw positions and preservation of the bony endplate were confirmed via micro-CT immediately after surgery (Fig. 7f).

Analgesics (2.5 mg/kg butorphanol; Meiji Seika Pharma Co., Ltd.) and antibiotics (22,000 units/kg 1% penicillin; Meiji Seika Pharma Co., Ltd.) were subcutaneously administered for 2 postoperative days. Animals were euthanized with carbon dioxide at 6 weeks postoperation, and the operated caudal segments were fixed with 10% formalin.

### In vivo* micro-CT analysis*

In vivo micro-CT was performed immediately after surgery, on postoperative day 2, and every week after surgery until euthanasia (6 weeks). The treated caudal vertebrae were scanned using micro-CT (R_mCT; Rigaku Mechatronics, Tokyo, Japan) at a resolution of 59 μm/voxel in vivo, and the data were collected at 90 kV and 160 μA. Visualization and data reconstruction were conducted using TRI/3D-BON (Ratoc System Engineering, Tokyo, Japan).

To quantify the soft tissue swelling at the surgical sites, the TV was calculated by setting a ROI on micro-CT images as follows: a rectangle including the skin surfaces in the axial width and 5 mm in the longitudinal length that centers the intervertebral disc space (threshold [L = 15500], software; TRI/3D-BON, Ratoc System Engineering, Tokyo, Japan) (Fig. 4a). The ratio of soft tissue swelling was calculated by dividing the TV of the surgical site on postoperative day 2 by the TV, 1 day before surgery.

### Ex vivo* micro-CT analysis*

Spinal segments harvested at 6 weeks postoperation were scanned using high-resolution ex vivo micro-CT (Skyscan 1272, Bruker, Belgium). The scanning parameters were as follows: camera binning = 2 × 2, source voltage (kV) = 80, source current (μA) = 125, image pixel size (μm) = 10, rotation step (degree) = 1.0, and filter = Al 1 mm. Image analysis was performed using CTAN software (Version 1.18.8.0 + , Bruker, Belgium).

### Histological analysis

Dissected and formalin-fixed caudal segments were fixed with 10% formic acid, dehydrated in a graded ethanol series, decalcified with K-CX (Falma, Tokyo, Japan), and embedded in paraffin wax. Serial sagittal Sects. (3 µm thickness) were cut and stained with H&E. A 1.5 × 2 mm^2^ ROI (interbody space) was extracted from the newly formed fusion mass. The new bone area (red) was color coded using the ImageJ software (version 1.52q, U. S. National Institutes of Health, Bethesda, Maryland, USA)^31^, and the percentage of the newly formed bone area in the ROI was calculated (Fig. 6a).

### Statistical analysis

All statistical analyses were performed using the JMP 15 Statistics software (SAS Institute Inc., Cary, NC, USA). Results are presented as means ± standard deviation. Fisher’s exact test was used to compare the success of fusion and the incidence of adverse events in each group. Differences in the measured variables between multiple groups were analyzed using one-way analysis of variance followed by Dunnett’s test or Tukey–Kramer test. Statistical significance was set at *p* < 0.05.

## Supplementary Information

Supplementary Information.

## Data Availability

The data that support the findings of this study are available from the corresponding author, TK, upon reasonable request.
