# Supplementary material for: Effects of rhBMP-2-loaded hydroxyapatite granules/beta-tricalcium phosphate hydrogel (HA/β-TCP/hydrogel) composite on a rat model of caudal intervertebral fusion
Source: Sci Rep. 2022 May 12;12:7906. doi: 10.1038/s41598-022-12082-y (PMC9098867; doi:10.1038/s41598-022-12082-y)
Supplement: Supplementary file 1 — Supplementary Information. [file 41598_2022_12082_MOESM1_ESM.pdf]

Effects of rhBMP-2 loaded hydroxyapatite granules/beta-tricalcium phosphate hydrogel (HA/β-TCP hydrogel) composite on a rat model of coccygeal intervertebral fusion

Shinichi Nakagawa[1], MD, Rintaro Okada[2], MD, PhD, Junichi Kushioka[1], MD, PhD, Joe Kodama[3], MD, Hiroyuki Tsukazaki[3], MD, Zeynep Bal[1], PhD, Daisuke Tateiwa[1], MD, Yuichiro Ukon[1], MD, Hiromasa Hirai[1], MD, Takahiro Makino[1], MD, DMSc, Shota Takenaka[1], MD, DMSc, Seiji Okada[1], MD, PhD, Takashi Kaito[1]*, MD, PhD

[1]Department of Orthopaedic Surgery, Osaka University Graduate School of Medicine, Suita, Osaka, Japan

[2]Department of Orthopaedic Surgery, Minoh Municipal Hospital, Minoh, Osaka, Japan

[3]Department of Orthopaedic Surgery, Kansai Rosai Hospital, Amagasaki, Hyogo, Japan

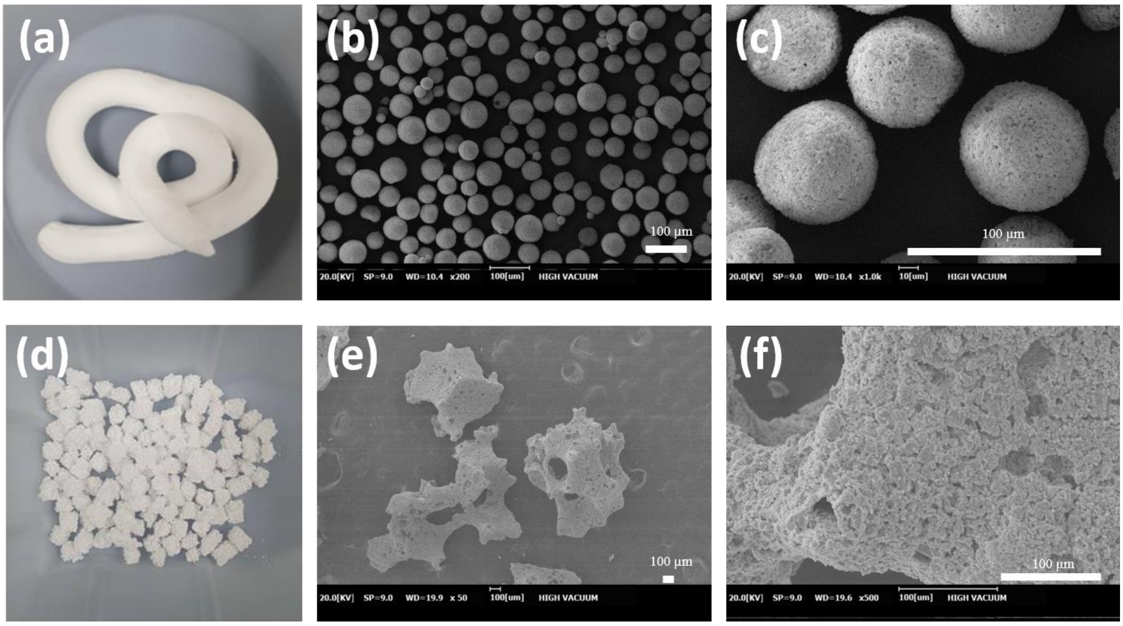

Supplementary Figure 1.

Macroscopic images (a; β-TCP hydrogel, d; HA) and two microstructural images of β-TCP hydrogel (b, c) and HA (e, f) with different magnifications; ×200 (b) and ×1000 (c), and ×50 (e) and ×500 (f) (bar; 100 µm).
